# Factors associated with patient willingness to participate in anaesthesia clinical trials: a vignette-based cross-sectional study

**DOI:** 10.1186/s12874-020-00949-5

**Published:** 2020-03-19

**Authors:** Caroline Noirmain, Béatrice Gil-Wey, Isabelle Pichon, Pauline Brindel, Guy Haller

**Affiliations:** 1 Division of Anesthesia, Department of Acute Care Medicine, Geneva University Hospitals and Faculty of Medicine University of Geneva, Rue Perret-Gentil 4, Geneva, Switzerland; 2 Clinical Research Center and Division of Clinical Epidemiology, Department of Health and Community Medicine, University of Geneva, University Hospitals of Geneva, Geneva, Switzerland; 3 Health Services Management and Research Unit, Department of Epidemiology and Preventive Medicine, Monash University, The Alfred Centre, 99 Commercial Road, Melbourne, Vic 3004 Australia

**Keywords:** Anesthesia, Clinical trials, Willingness to participate, reasons for refusal

## Abstract

**Background:**

Clinical trials are essential to improve knowledge of anesthesia and perioperative medicine. Unfortunately, many studies face participant-recruitment issues and fail to include the planned number of participants. There is limited published data about how information delivered about the study or how the experiences and attitudes of prospective participants influence willingness to participate. The purpose of this study was to identify such factors in the domain of anesthesia care.

**Methods:**

We performed a cross-sectional study at the Geneva University Hospitals (Switzerland) using a newly developed paper-based questionnaire on a sample of outpatients with a recent hospital stay and that were aged over 18 years, confident speaking French and free of any disease that could hinder participation. We explored patient personal factors, such as current health, past exposure to clinical research and anesthesia, as well as study-related factors. Six different scenarios for clinical studies were assessed. Linear regression modeling was used to assess the specific association between personal and study-related factors and willingness to participate in the studies described in the scenarios.

**Results:**

On the 1318 eligible patients, 398 fully completed the questionnaire. Multivariable adjustment revealed that factors related to altruistic values (*β*, 9.6, 95% CI 3.4 to 15.7, *P* = 0.002), to the feeling of benefiting from a more effective treatment (*β*, 4.7, 95% CI 0.2 to 9.2, *P* = 0.041) and to the absence of fear about double blinding (*β*, 5.7, 95% CI 1.3 to 10.2, *P* = 0.012) were positively associated with willingness to participate. Conversely, concerns about drug-related adverse effects (*β*, − 11.7, 95% CI − 16.9 to − 6.5, *P* < 0.001) and anxiety about surgery (*β*, − 5.2, 95% CI − 10.0 to − 0.5, *P* = 0.031) were negatively associated with willingness to participate.

**Conclusion:**

Our study was based on vignettes illustrating typical scenarios of clinical trials performed in anesthesia. However, their similarities with real studies still remains hypothetical and our results should be interpreted as such. Nevertheless, the study contributes to improve understanding of factors that may act as incentives or barriers to participation in clinical trials. It highlights the importance of providing appropriate information and reassurance to patients.

## Background

Clinical trials inform clinical practice and contribute to its improvement [1]. However, numerous clinical trials are not published because they fail to recruit the planned number of participants [2, 3]. A recent publication revealed that more than 17.2% of the clinical trials accepted by Ethics Committees had been abandoned [4]. A study conducted in the United Kingdom revealed that only 31% of approved clinical trials had completed participant recruitment on time and with the planned number of participants [5]. In 42% of the clinical trials, the number of participants had to be modified, resulting in sample number reduction for most studies and, thus, a loss of study power. Finally, it was also demonstrated that 54% of trials had an unplanned extension of the duration of the study, resulting in increased study costs [5].

A lack of rigor in managing and finalizing clinical research projects is often attributed to participant recruitment issues [6]. Several factors negatively influencing willingness to participate have previously been identified, including patient fear of side effects or of receiving a less effective treatment [7, 8], a poor understanding of the randomization or blinding process or of the placebo treatment principle [9, 10], or patient a priori preference for a specific treatment [11]. In addition, studies requiring additional testing, such as blood sampling [12], an extra visit [13, 14] or long commuting to the study site [15], are also less likely to recruit patients. Finally, patients can also develop distrust of study investigators and refuse to participate if they do not understand the research subject [16], develop the feeling of being a “guinea pig” [17] and fear that the treatment is beyond their control [18, 19]. Most of these barriers to patient participation in clinical trials were identified from studies in the field of cancer medicine [20–22] or infectious disease [23, 24].

While challenges for participant recruitment are also encountered in the field of anaesthesia [7, 25], the evidence in the literature about the factors influencing participation is still limited in the field. Early studies in anesthesia performed in the United States and Canada in the context of same-day surgery demonstrated that patient participation in a clinical trial can be influenced by the timing of the first encounter with investigators [26]. It has also been shown that the use of invasive procedures (i.e., bronchoscopy, epidural catheter insertion) can significantly decrease study participation (43 to 67%) [27, 28]. Apart from these limitations, it currently remains largely unclear which patient personal characteristics and/or study-related factors are associated with refusal or acceptance for participation in clinical trials for anesthesia. Therefore, we conducted a cross-sectional study based on a paper-based questionnaire to specifically assess the attitudes of inpatients towards anesthesia clinical trials.

## Methods

### Design and setting

The study was conducted between 2013 and 2014 at the Geneva University Hospitals (Switzerland) a tertiary teaching hospital network of 1835 beds that includes all specialties (pediatrics, geriatrics, psychiatrics, medicine and surgery). The protocol was approved by the Central Ethics Committee of Human Research of the Geneva University Hospitals, Switzerland (CER 12–123 – NAC 12–049). A waiver for explicit written individual consent was provided by the ethics committee for this questionnaire study.

### Questionnaire content and administration

The questionnaire was developed following an extensive literature review [29–34] and meetings with experienced clinical researchers at the hospital. Their professional expertise was used to identify and classify the most meaningful factors from the literature that may hinder patient participation to clinical trials. We identified 18 personal and study-related factors likely to influence patient participation and were included in the questionnaire. Personal factors included beliefs about best care practice, altruism, previous participation in a clinical trial, level of trust in healthcare professionals, and fears of side effect from medication and procedures. We explored opinions on ways to improve medical knowledge, possible benefits of new treatments, beliefs of individual patients and overall stress associated with surgery and medication. Study-related factors included financial compensations for study participants, commuting to the clinical trial, blood test results, randomization, double-blinding (neither investigators nor participants are aware of the type of treatment received), and the type of “placebo” treatment used. We also assessed general knowledge and attitudes towards clinical research as well as demographic characteristics and health conditions of potential participants.

Clinical trials in anesthesia aim at optimizing the management of patient through therapeutic test or investigations. In our study, the willingness to participate in a clinical trial was assessed using six different types of scenarios related to anesthesia care and that included: 1) a new drug not currently available on the market, 2) a new dosage of a well-established medication, 3) a new indication of a current medication, 4) side effects of a well-established medication, 5) a new locoregional anesthesia technique (i.e., epidural or nerve block), and 6) a new anesthetic monitoring device (i.e., blood pressure measurement, electrocardiogram). For each proposed scenario, participants were asked about their willingness to participate to the type of study described in the context of anesthesia care.

Answers were rated on a 5-point Likert scale, including the following choices: “I would certainly accept”, “I would probably accept”, “I don’t know if I would accept”, “I would probably refuse”, “I would certainly refuse”. The final questionnaire (provided in Additional file 1) included 46 questions and was pre-tested for understanding and formatting on a convenience sample of 10 volunteers.

A random group of 1800 patients aged over 18 years, fluent in French, free of any severe disease hindering participation (i.e., cognitive disorder, severe handicap) and admitted to the Geneva University Hospitals within the three previous years was identified in the hospital administrative database and listed for the study. As the data did not allow the identification of non-eligible patients, they all received a questionnaire sent by post mail. However, the questionnaire was accompanied by an introduction letter explaining the study purpose and allowed participants to return an empty questionnaire if they felt they could not answer because: “they were not confident speaking French”, “had poor health condition”, “could not participate in any type of study” and “had other reason” with the space to provide comments. In addition patients who had died since their hospital stay were identified in the death registry or through contact with families.

The questionnaire was designed to protect the anonymity of the respondents. A unique ID number was assigned to each questionnaire sent. Respondents’ names were only available to one research nurse in charge of mailing the questionnaires and reminders.

### Sample size calculation and statistical analysis

A power calculation was performed to obtain a sample size able to detect a significant difference in the level of participation barriers according to willingness to participate in a study, with an α error of 0.05 and a study power of 0.90. A published study [35] in the field of oncology demonstrated that the mean and standard deviation (SD) of participation barriers scores for patients willing and unwilling to participate were 21.19 ± 7.27 and 23.78 ± 7.15, respectively. The calculated study sample of 328 participants was increased to 1800 patients accounting for non-eligibility and an expected low response rate.

For descriptive analysis of participant personal factors, we used frequencies, proportions and means with SD. The six different scenarios assessing willingness to participate in a clinical study were tested for correlations and internal consistency with Spearman correlation and Cronbach’s alpha statistic. A value of Cronbach α ≥0.7 was considered adequate. Cronbach’s alpha analysis indicated a reliability of 0.84, corresponding to a correlation of √0.84 = 0.92 between the measured scale and the underlying factor. Moreover, our results from factor analysis indicated that one main factor was meaningful. To be retained, a factor needed to have an eigenvalue over 1. Since a single underlying dimension was identified (Table 1), the 6 scenario variables were combined into a unique standardized score. The analysis showed that a single underlying dimension could be identified, common to the 6 scenarios that were named “willingness to participate”. We considered that the underlying concept of willingness to participate was continuous and that the intervals between the different answers on the Likert scale were equal. For each of the scenario a subscore was calculated, using Mean and Standard Deviation (SD). A scale from 0 to 100 was created by averaging the sum of all individual item scores, standardizing and reversing the scoring as it had negative correlations with the factor being measured. We used the following formula: 25*(5-score). As a result, we created a score on a continuous scale of 0 to 100, with 100 corresponding to the highest level of willingness and 0 to the lowest level of willingness to participate to a clinical trial. “No opinion” was considered as a missing value.

**Table 1:** Factorial analysis and factor loadings

Scenarios	Factor 1	Factor 2	Factor 3	Uniqueness
New drug not currently available	0.68	−0.19	0.05	0.50
New dosage of a well-established drug	0.81	−0.03	− 0.07	0.34
New indication of a current medication	0.82	−0.04	−0.08	0.33
New side effect of a well-established drug	0.59	0.15	−0.09	0.63
New locoregional anesthesia technique	0.63	0.03	0.15	0.57
New anesthetic monitoring device	0.57	0.14	0.09	0.64
Eigen value	2.85	0.08	0.05	

To measure the association between patient personal characteristics, past exposure to clinical research, personal or study-related variables and willingness–to-participate scores, univariate analysis was performed. Differences in mean scores according to the different characteristics or reasons considered were compared using Student’s t test, the Mann-Whitney-Wilcoxon test for binary factors, analysis of variance, or, for more than 2 factors, Kruskall-Wallis test, depending on distribution. A non-parametric test for trend (Cuzick test) was applied to compare mean scores of willingness to participate across ordered groups, corresponding to levels of response to questions about personal reasons to participate and study-related factors. A *P* value less than 0.05 was considered significant**.** Stepwise regression analysis was performed using a backward modeling procedure to regress the willingness–to-participate score on the reasons to participate, adjusted for patient personal characteristics and past exposure to research that were significantly associated in univariate analysis. Variables corresponding to personal reasons to participate and study-related factors were recoded as binary variables before inclusion in the model. The distribution of residuals was checked for all linear regression models*.* Analyses were performed using Stata 15 statistical software (StataCorp LLC, College Station, TX, USA).

## Results

**Fig. 1:**
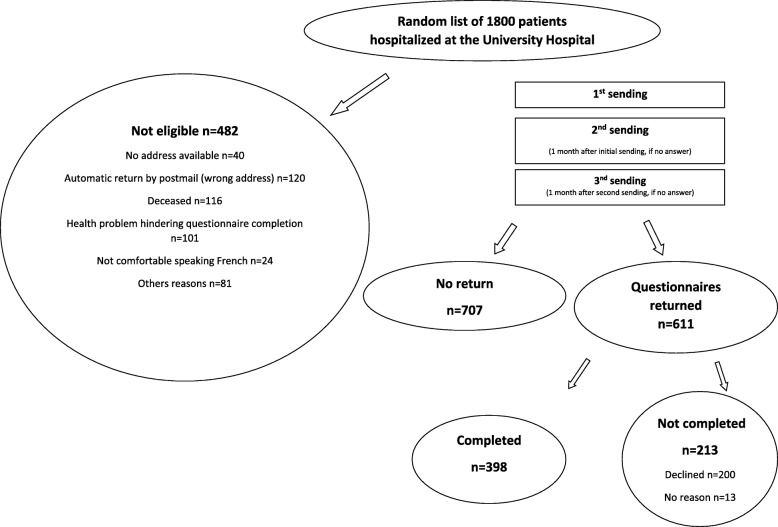
Flow chart of study process

Figure 1 depicts the study flow chart. From the initial random list of potential recipients, 482 patients were considered as non-eligible because they were deceased (*N* = 116), had a wrong postal address, had moved abroad or were in psychiatric institution or prison (*N* = 160), had health problem hindering questionnaire completion (*N* = 101), were not comfortable speaking French (*N* = 24) or had other reasons (*N* = 81). Of the 1318 remaining eligible patients, 611 returned the questionnaire (absolute contact rate 46%) and 398 of these fully completed the questionnaire (65% absolute cooperation rate). A total of 213 recipients either returned a blank questionnaire or expressed their unwillingness to participate. There was no evidence for a difference in gender distribution (*P* = 0.96) or an age difference for patients who declined to participate in the study compared with those who participated (60.0 ± 20.9 vs 59.5 ± 19.8 years; *P* = 0.65).

### Factor analysis results

The inter-item correlation matrix for the six scenarios showed correlation coefficients ranging between 0.33 and 0.71. Cronbach’s alpha analysis indicated a good level of internal consistency for the different scenarios (α = 0.84). Following factor analysis, one main factor (Factor 1) appeared to be meaningful, with factor loadings for the different scenarios ranging between 0.57 and 0.82. (Table 1). This factor was considered to describe patient willingness to participate in research as it correlated positively with all six scenarios. Uniqueness (the proportion of variance for a variable that is not explained by the different factors) was significant for scenarios referring to a study on side effects of drugs and a study evaluating a new monitoring device (> 0.6). Distribution of the score of willingness to participate across the six scenarios was approximately Gaussian. Mean score was 59.9 ± 19.6.

### Patient characteristics

The majority of participants were female *N* = 208 (54.7%) and were aged over 59 years. A large number of participants (40.1%) had received short cycle tertiary education or more (Table 2). Univariate analyses revealed significantly higher mean scores of willingness to participate (*P* < 0.05) for males, respondents regularly taking medications and respondents with a past experience in anesthesia and clinical research (Tables 2 and 3).

**Table 2:** Univariate analysis for mean score of willingness to participate in a clinical study in anesthesia, according to patient characteristics

Patient characteristics	N (%)	Mean score of willingness to participate ± SD	***P*** value
Sex			
Male	172 (45.3%)	63.3 ± 18.2	0.002*
Female	208 (54.7%)	57.1 ± 20.3	
Age (year)			
< 30	27 (7.2%)	60.0 ± 17.0	0.222
30–39	52 (13.9%)	56.3 ± 20.0	
40–49	51 (13.6%)	59.5 ± 24.7	
50–59	65 (17.3%)	60.2 ± 17.3)	
60–69	82 (21.9%)	60.1 ± 20.9	
70–79	63 (16.8%)	63.1 ± 17.5	
≥ 80	35 (9.3%)	59.9 ± 18.3	
Education			
Basic education	44 (11.8%)	57.4 ± 17.0	0.63
Apprenticeship	97 (26.1%)	62.8 ± 17.8	
Upper secondary education	25 (6.7%)	60.0 ± 18.1	
Short cycle tertiary education	72 (19.4%)	60.2 ± 18.6	
Bachelor or higher university degree	77 (20.7%)	58.1 ± 22.5	
Other	57 (15.3%)	59.6 ± 22.7	
Professional activity			
Full-time employment	71 (18.9%)	60.0 ± 18.1	0.42
Part-time employment	64 (17.1%)	56.1 ± 18.7	
Working at home	16 (4.3%)	60.7 ± 22.9	
Unemployed	25 (6.7%)	64.7 ± 23.0	
Student	11 (2.9%)	61.7 ± 11.5	
Retired or invalid	188 (50.1%)	59.9 ± 20.0	
Working in health care			
Yes, medical	41 (10.9%)	63.1 ± 23.2	0.38
Yes, other	37 (9.8%)	58.3 ± 20.8	
No	299 (79.3%)	59.7 ± 19.0	
Chronic disease			
Yes	151 (42.5%)	60.8 ± 20.7	0.35
No	204 (57.5%)	58.9 ± 19.0	
Taking medication			
Yes	258 (68.8%)	62.2 ± 19.6	0.0004
No	117 (31.2%)	54.5 ± 19.2	
Past hospitalization			
Yes	367 (97.6%)	60.3 ± 19.4	0.25
No	9 (2.4%)	52.8 ± 18.6	

*T-test for gender, chronic disease, medication; Wilcoxon test for hospitalization and anesthesia; ANOVA for education; Kruskall Wallis test for professional activity and health sector, Chi-square test for trend for age

**Table 3:** Past exposure to clinical research and mean scores for willingness to participate in a clinical trial

Past exposure to clinical research	n (%)	Mean score of willingness to participate ± SD	***P*** value
Have you ever been invited to participate to a clinical study in any medical area?			0.05*
No	232 (64.1%)	58.1 ± 19.9	
Yes	130 (35.9%)	62.4 ± 19.6	
Have you ever been invited to participate to a clinical study in the area of anesthesia care?			0.09*
No	9 (7.1%)	50.0 ± 24.5	
Yes	117 (92.9%)	64.0 ± 18.6	
Have you ever heard about clinical research involving inpatients?			0.0054*
Yes	245 (65.7%)	61.8 ± 20.1	
No	128 (34.3%)	55.9 ± 18.3	
If yes, who informed you about clinical research involving inpatients?			
Media	120 (49.0%)	60 ± 20.4	†
Family	22 (9.0%)	59.5 ± 18	
Friends	35 (14.3%)	58.3 ± 19.9	
Family doctor	29 (11.8%)	67.8 ± 15.3	
Hospital doctor	117 (47.8%)	62.9 ± 20.2	
Other	38 (15.5%)	62.2 ± 21.9	

* Student’s T-test

†*P* value not available, multiple answers possible for this questio

### Personal and study-related factors

Table 4 summarizes the personal factors that were significantly associated with willingness to participate to clinical trials. The majority of patients exhibiting altruistic values (desire to help others or to contribute to medical progress) or with a strong perception to benefit from a more effective or better treatment, exhibited higher mean scores for willingness-to-participate (MWS) (*P* < 0.001). In contrast, lower willingness-to-participate scores were observed in a significant proportion of patients who were concerned about drug side effects (MWS 48.5 ± 20.8) or inefficacy (MWS 47.9 ± 25.3) as well as those who reported distrusted doctors (MWS 38.9 ± 29.0) (Table 4).

**Table 4:** Personal factors and mean willingness-to-participate scores

Personal factors influencing the willingness to participate	Strongly agreed	Agreed	Indifferent	Disagreed	Strongly disagreed	***P*** value*
I could receive better treatment. N (%)	65 (18.5%)	182 (51.9%)	52 (14.8%)	34 (9.7%)	18 (5.1%)	
Mean score ± SD	69.6 ± 19.0	60.9 ± 17.6	58.7 ± 18.3	56.8 ± 21.3	50.7 ± 24.5	< 0.001
I could help other patients. N (%)	110 (32.5%)	179 (52.8%)	33 (9.7%)	13 (3.8%)	4 (1.2%)	
Mean score ± SD	67.8 ± 17.4	60.3 ± 17.9	52.5 ± 21.3	44.9 ± 19.4	40.6 ± 18.8	< 0.001
I could contribute to medical progress. N (%)	147 (42.0%)	172 (49.1%)	23 (6.6%)	6 (1.7%)	2 (0.6%)	
Mean score ± SD	68.9 ± 16.3	57.4 ± 18.3	51.4 ± 22.0	45.8 ± 19.7	33.3 ± 11.8	< 0.001
I would be considered a “guinea pig.” N (%)	34 (9.8%)	100 (28.9%)	100 (28.9%)	55 (15.9%)	57 (16.5%)	
Mean score ± SD	50.1 ± 24.1	56.4 ± 18.0	63.7 ± 16.9	62.5 ± 15.4	70.5 ± 20.3	< 0.001
I trust doctors. N (%)	83 (23.2%)	204 (57.0%)	40 (11.2%)	25 (7.0%)	6 (1.7%)	
Mean score ± SD	68.2 ± 18.4	60.7 ± 17.0	54.7 ± 17.1	51.7 ± 23.8	41.0 ± 28.6	< 0.001
I am afraid of adverse events related to the drug tested. N (%)	82 (23.3%)	155 (44.0%)	52 (14.8%)	47 (13.4%)	16 (4.6%)	
Mean score ± SD	48.5 ± 20.8	58.2 ± 16.7	70.1 ± 16.5	69.8 ± 14.7	72.6 ± 26.9	< 0.001
I could receive a more effective treatment. N (%)	62 (18.7%)	159 (47.9%)	57 (17.2%)	42 (12.7%)	12 (3.6%)	
Mean score ± SD	70.1 ± 16.9	62.7 ± 16.1	56.9 ± 20.2	53.8 ± 18.5	54.5 ± 29.8	< 0.001
I would respect my personal convictions. N (%)	84 (25.5%)	126 (38.3%)	100 (30.4%)	12 (3.7%)	7 (2.1%)	
Mean score ± SD	65.6 ± 18.9	62.8 ± 15.7	59.5 ± 19.9	49.7 ± 19.2	46.4 ± 28.4	< 0.001
I would be anxious about surgery. N (%)	60 (17.0%)	130 (36.8%)	68 (19.3%)	63 (17.9%)	32 (9.1%)	
Mean score ± SD	52.4 ± 24.1	56.7 ± 18.0	64.2 ± 16.8	64.1 ± 14.9	76.5 ± 18.0	< 0.001
I would be worried about inefficacy of the drug tested. N (%)	48 (14.1%)	119 (35.0%)	73 (21.5%)	76 (22.4%)	24 (7.1%)	
Mean score ± SD	47.9 ± 25.3	57.2 ± 16.6	61.6 ± 16.5	66.8 ± 18.1	80.5 ± 17.3	< 0.001
I think that research only benefits the doctor’s career. N (%)	12 (3.5%)	29 (8.3%)	38 (10.9%)	125 (35.9%)	144 (41.4%)	
Mean score ± SD	38.9 ± 29.0	55.7 ± 14.8	57.0 ± 12.9	59.3 ± 17.7	65.9 ± 19.5	< 0.001

*Cuzick test for trend

Study-related variables also had significant influence on willingness to participate (Table 5). Studies requiring a blood test or travel were considered as less attractive (MWS 44.3 ± 21.5 and 48.3 ± 25.2, respectively). Randomization was considered by 58.5% of the patients as a significant disincentive to participate in clinical research (MWS 50.4 ± 22.6, *P* < 0.001), whereas double blinding (MWS 48.5 ± 23.8, *P* < 0.001) and the use of a placebo (MWS 43.2 ± 27.8, *P* < 0.001) were considered as problematic by less than half of the patients (44.3 and 28.8%, respectively). In contrast, financial compensation for participation was significantly associated with a higher willingness to participate (*p* = 0.006).

**Table 5:** Study-related factors and mean willingness-to-participate scores

Study-related factors influencing the willingness to participate	Strongly agreed	Agreed	No opinion	Disagreed	Strongly disagreed	***P*** value*
If you received financial compensation, would you be more willing to participate? N (%)	55 (14.9%)	62 (16.8%)	70 (18.9%)	73 (19.7%)	110 (29.7%)	
Mean score ± SD	65.5 ± 17.6	62.2 ± 15.8	63.1 ± 16.2	56.2 ± 18.8	57.3 ± 23.6	0.006
If participating in the study required commuting to the hospital, would you decline to participate? N (%)	40 (11.1%)	79 (21.9%)	57 (15.8%)	111 (30.8%)	74 (20.5%)	
Mean score ± SD	48.3 ± 25.2	56.6 ± 17.3	60.8 ± 17.8	62.3 ± 15.5	69.1 ± 19.6	< 0.001
If the study required blood tests, would you decline to participate? N (%)	24 (6.4%)	46 (12.3%)	52 (13.9%)	110 (29.4%)	142 (38.0%)	
Mean score ± SD	44.3 ± 21.5	55.5 ± 17.7	55.4 ± 19.2	58.6 ± 15.1	68.2 ± 19.1	< 0.001
If the study required randomly assigned treatment, would you decline to participate? N (%)	95 (27.1%)	110 (31.4%)	42 (12.0%)	76 (21.7%)	27 (7.7%)	
Mean score ± SD	50.4 ± 22.6	58.6 ± 15.2	64.4 ± 16.4	66.1 ± 16.4	69.1 ± 24.4	< 0.001
If the study required a treatment that was prescribed by the study and not by your doctor, would you be willing to participate? N (%)	37 (10.4%)	143 (40.1%)	39 (10.9%)	84 (23.5%)	54 (15.1%)	
Mean score ± SD	71.1 ± 21.6	64.9 ± 13.3	59.5 ± 15.5	54.4 ± 19.3	48.4 ± 26.5	< 0.001
If the study involved a placebo (inactive substance), would you decline to participate? N (%)	40 (11.3%)	62 (17.5%)	65 (18.3%)	101 (28.5%)	87 (24.5%)	
Mean score ± SD	43.2 ± 27.8	56.7 ± 18.0	60.6 ± 17.3	61.2 ± 15.9	68.5 ± 17.2	< 0.001
Many clinical trials are double-blind studies. This means that neither doctors nor the participants are aware which participants receive the test drug and which receive the placebo. Would you consider this a reason to decline participation? N (%)	62 (17.5%)	95 (26.8%)	34 (9.6%)	82 (23.2%)	81 (22.9%)	
Mean score ± SD	48.5 ± 23.8	54.5 ± 16.6	64.9 ± 12.2	64.8 ± 16.0	70.4 ± 19.1	< 0.001

*Cuzick test for trend

### Independent personal and study-related factors predicting willingness to participate

Personal and study-related factors were retained for the multivariable linear regression analysis of the score of willingness to participate in research and adjusted for gender, regular use of medication and past exposure to anesthesia care and clinical research. Factors that remained significant were those related to altruistic values “I could help other patients” (*P* = 0.02), “I could contribute to medical progress” (*P* = 0.012) and the expectation of benefiting from a more effective treatment (*P* = 0.041). Among other personal factors, worries about adverse events related to the drug tested (*P* < 0.001) and worries related to surgical intervention (*P* = 0.031) had a significant negative impact on the willingness to participate. The only study-related factor that was associated with willingness to participate was double blinding (*P* = 0.012) (Table 6). Patients who did not consider it as an issue were significantly more likely to participate.

**Table 6:** Multivariable logistic-regression model predicting willingness to participate

	Willingness to participate in a clinical trial Beta coefficient	95% Confidence interval	*P* value
Intercept **(alpha)**	30.2	13.3 to 47.1	0.001
I am afraid of adverse events related to the drug tested (yes/ no)	−11.7	−16.9 to −6.5	< 0.001
I could help other patients. (yes/ no)	9.6	3.4 to 15.7	0.002
I would not consider double-blinding a reason to decline participation. (yes/ no)	5.7	1.3 to 10.2	0.012
I would be anxious about surgery.	−5.2	−10.0 to −0.5	0.031
I could contribute to medical progress. (yes/ no)	9.5	2.1 to 17.0	0.012
I could receive a more effective treatment. (yes/ no)	4.7	0.2 to 9.2	0.041

## Discussion

Assessing different kind of hypothetical scenarios of clinical trials in anesthesia, the present study identified a number of personal and study-related factors that were significantly associated with a likely willingness to participate in clinical trials for anesthesia research. Factors related to altruistic values, the expectation of beneficiating from a more effective treatment and the absence of worries about double blinding were positively associated with willingness. Conversely, concerns about adverse events related to the test drug and worries about surgery had a negative impact on willingness.

In line with a previous publication by Lobato et al. [36], we found that altruism and the feeling of contributing to the progress of science were significant incentives for patients to enroll in a clinical trial. This can be explained by the intrinsic nature of altruism, a behavior that leads to prefer benefits to third parties rather than personal advantages. Like fire-fighters who can put their own life or health in danger to help their community [37], patients with altruistic values are more likely to accept to enroll in a clinical trial and to take the risk of testing a new medication or technique if a benefit for the community is perceived.

Another factor identified in the present study is the expectation that participating in a study is a way of improving personal fitness and benefiting from a better medical treatment. Such expectations have been reported to increase willingness to participate by 4.7 fold, and implied a feeling of benefiting from special attention if recruited in a trial [38]. Similarly, it has been shown that patients with terminal cancer favored study protocols offering curative over palliative treatments, even if the study included invasive procedures such as biopsies [39]. Patients with precarious health are therefore more likely than healthy patients to be willing to participate in clinical trials. They can perceive an additional benefit of an experimental drug over traditional therapy. Paradoxically most study tend to exclude patients with severe comorbidities, as they are expected to experience more often possible side effects of new treatments tested.

A highly significant disincentive to participation was related to inconveniences, including travel to the study site and invasive procedures such as blood-sampling. This finding is confirmed by previous studies [12, 39, 40]. Patients’ perception of additional risk or pain resulting from participation to a trial had a negative impact on willingness to participate [40]. The same phenomenon has been identified in pediatric studies [41].

Concern about risk is of particular interest and constitutes a controversial aspect of study inclusions. The principle of patient autonomy, largely supported by ethics committees, enforces that potential participants have full knowledge of the risks and benefits of study participation in order to guarantee informed consent. In Switzerland, patient information is also regulated by a federal law that requires healthcare professionals to prove that they have provided enough information before any medical intervention or treatment (experimental or usual) that guarantees patient informed consent [42]. However, while disclosing an exhaustive list of all possible risks associated with a treatment (or in the case of a clinical trial, with study participation) can be considered as a recommended approach to ensure informed consent, this may also cause a lot of anxiety. Potential benefits of participation might be disregarded when patients are first presented with a long list of potential risks resulting in a high refusal rate, despite short or long term possible benefits.

Current literature suggests alternative approaches to ensure informed consent based on more personalized information processes. Mingus et al. for instance, found that patients who were approached by their own physician, who used open-ended questions and addressed more specifically patients’ personal concerns, were more likely to participate to a clinical trial [25]. A similar finding was reported in a pediatric study [43]. These findings emphasize that willingness to participate is strongly influenced by trust, confidence and the feeling of familiarity with the different aspects of study content, the latter of which is essential to guarantee informed consent [44]. The potential disincentive of full disclosure of risks can potentially be mediated by ensuring patients in the recruitment process are provided with individual attention by health care professionals that are willing and qualified to listen carefully to patient concerns and explain the details to a depth that satisfies the patient.

Another interesting finding in the present study is that the lack of fear about double-blinding was positive predicting factor for participation. This result agrees well with current literature reporting that specific procedures associated with trial design, such as randomization, the use of a placebo or double-blinding, are often poorly understood by patients and can become significant barriers to participation [45]. These findings emphasize again that patients must be provided the required information, including complex concepts such as double blinding, in a way that they can understand sufficiently for informed consent.

We also found that patients taking medication on a regular basis were more willing to participate. This may be explained by the fact that taking medication on a regular basis is a surrogate of having a chronic disease. As identified in the study, a majority of patients believe they can benefit from a better treatment when participating to a trial. It is quite likely that both items are correlated and that patients with chronic disease (taking medication on a regular basis) are more willing to participate because they believe they could benefit from a better treatment and recover from their disease.

A number of limitations of this study need to be mentioned. One is the use of a randomization process to identify on a list of discharged hospital patients, potential study participants. While this ensured a representative sample of in hospital patients likely to be enrolled in clinical trials, we were unable to fully identify before sending the questionnaires, patients who would obviously be unable to participate. This included deceased patients, those having language barriers hindering study participation and those unable to answer due to severe disease. This resulted in a significant number of patients being approached (*N* = 482) who should have been excluded at study beginning. Despite these limitations, our population sampling presented a good cooperation rate (65%) allowing significant and valid conclusions.

Another limitation is the non-participation of patients with severe health problems. As their willingness to participate could not be established, we are unable to draw valid conclusions on this category of patients regarding their willingness to participate to a trial in the area of anesthesia care. At the same time, we still included a significant number of participants (68.8%) reporting regular use of medication for a chronic disease, tempering the non-participation of patients with severe health problems.

Another limitation is the use of hypothetical scenarios of anesthesia trials to assess willingness to participate. This methodological approach may increase the risk of information bias since a number of participants may not fully understand the exact content of the vignettes provided. This may distort their answers and their ability the reliably identify true differences between the different scenarios provided. However, clinical vignettes are common tools used in studies assessing patient perspective on the qualitative dimensions of medical practice and research. They can significantly contribute to improve understanding of patient perceptions on these topics.

Finally, the questionnaire addressed issues that had for some of them, been previously identified as factors influencing willingness to participate in a clinical trial. In addition the exclusive use of closed-ended questions in our questionnaire allowed only quantitative assessment. As a result we could not identify the full range of possible barriers to clinical trial participation that a qualitative study would have allowed.

Despite these limitations, the present study provides valuable information about personal and study-related factors that influence patient willingness to participate to clinical trials in anesthesia care. While in any trial patients have to balance their willingness to participate according to a specific risk benefits ratio, knowing which factors are more likely to worry patients and ultimately lead to their refusal to participate may be very helpful. For instance, in a trial testing a new anesthetic drug, knowing patient’s fear of side effects of the medication provided may help to better detail these side effects (without minimizing them) and as a result decrease patient’s level of anxiety. In addition, explaining to patients the benefits of participation not only for them but also for the wider community may contribute to an enhanced level of participation in patients with altruistic values.

Therefore, we suggest that, to improve participation, researchers should ensure that required information is comprehensively explained to patients and that the important aspects of study participation are emphasized. The information should highlight personal and collective benefits resulting from the participation in a study, as well as clearly explaining the possible adverse effects of the treatment and thoroughly address patient concerns about any surgical intervention involved in the treatment. Information delivered to patients should be pertinent, understandable and address patients’ concerns in a way that builds trust. Healthcare organizations recruiting participants to clinical trials should consider the development of practical handbooks that alert both researchers and patients to key factors influencing willingness to participate. Future research should assess in a prospective study new approaches to improve participation rates by providing targeted information strategies in face-to-face encounters and that address patient attitudes and fears.

## Conclusion

The power of clinical trials can become compromised and costly when patient participation falls below expectations. Assessing different kind of hypothetical scenarios of clinical trials in anesthesia, the present study has identified a number of factors that may affect willingness to participate positively (altruism, benefits from a more effective treatment, absence of concerns about double blinding) and negatively (fear of adverse effects of the test drug, anxiety about surgery). It is hoped that, by taking these factors into consideration, clinical trial organizers can improve participation by providing required and appropriate information and reassurance to the patients.

## Supplementary information

**Additional file 1.** Questionnaire. Participation in a clinical trial in anesthesia: attitude, barriers and motivations of patients.

## Data Availability

The datasets generated and/or analysed during the current study are not publicly available due to absence of formal ethics approval for this, but are available from the corresponding author on reasonable request.

## Abbreviations

SD: Standard Deviation
MWS: Mean Scores for Willingness-to-participate
