# Supplementary material for: Factors associated with patient willingness to participate in anaesthesia clinical trials: a vignette-based cross-sectional study
Source: BMC Med Res Methodol. 2020 Mar 19;20:67. doi: 10.1186/s12874-020-00949-5 (PMC7082904; doi:10.1186/s12874-020-00949-5)
Supplement: Supplementary file 1 — Additional file 1. Questionnaire. Participation in a clinical trial in anesthesia: attitude, barriers and motivations of patients. [file 12874_2020_949_MOESM1_ESM.docx]

**
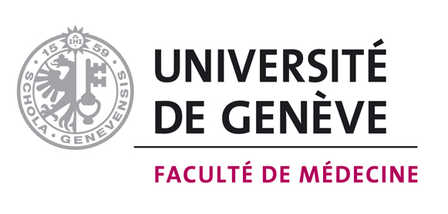
** [
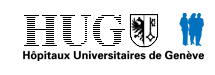
](http://www.hug-ge.ch/index.html)

**Participation in a clinical trial in anesthesia:**

**attitude, barriers and motivations of patients**

Patient survey

**New treatments are regularly developed and tested in clinical trial with volunteer patients. It is essential to better understand patients’ incentives to participate in clinical studies in the field of anesthesia. This is why we would like to invite you to participate to this study by answering to the questions below.**

**Instructions for completing this questionnaire:**

Please answer each question as closely as possible to your feelings. There are no right or wrong answers, only your personal experience matters. Total time to complete this questionnaire is approximately 15 minutes.

Your answers are important and will be managed in total anonymity.

**If you don’t want to answer to the questionnaire, please complete only this page and return the empty questionnaire to us with the stamped envelope attached.**

I am not confident speaking French

I have poor health condition

I could not participate in any type of study

other reason : ……………………………………………………………………………………………………

**Questionnaire**

**Question 1 : Have you ever heard about clinical research involving inpatients?**

_1_ yes _2_ no

If you answered “no” to question number 1 go directly to question 4.

If you answered “yes” continue with question 2.

**Question 2 : Who informed you about clinical research involving patients?**

(many answers are possible)

_1_ media

_2_ family

_3_ friends

_4_ family doctor

_5_ hospital doctor

_6_ other ………………………………………..

**Question 3 : Did this information reassure you?**

strongly rather indifferent rather strongly no

agree agree disagree disagree opinion

_1_ _2_ _3_ _4_ _5_ _6_

**Question 4 : Are you in favor of clinical research done in hospitals with patients?**

strongly rather indifferent rather strongly no

agree agree disagree disagree opinion

_1_ _2_ _3_ _4_ _5_ _6_

**Question 5 : Are you in favor of clinical research in anesthesiology?**

strongly rather indifferent rather strongly no

agree agree disagree disagree opinion

_1_ _2_ _3_ _4_ _5_ _6_

**Question 6 : Below are some reasons that may influence your decision to accept or refuse to participate in a clinical trial in the field of anesthesiology. For each proposition, please choose the answer that seems closest to your feelings.**

strongly rather indifferent rather strongly no

agree agree disagree disagree opinion

**a) I could receive better treatment** _1_ _2_ _3_ _4_ _5_ _6_

**b) I could help other patients** _1_ _2_ _3_ _4_ _5_ _6_

**c) I could contribute to medical**

**progress** _1_ _2_ _3_ _4_ _5_ _6_

**d) I would be considered as a**

**“guinea pig”** _1_ _2_ _3_ _4_ _5_ _6_

**e) I trust doctors** _1_ _2_ _3_ _4_ _5_ _6_

**f) I am frightened of adverse events**

**related to the drug tested** _1_ _2_ _3_ _4_ _5_ _6_

**g) I could receive a more effective**

**treatment** _1_ _2_ _3_ _4_ _5_ _6_

**h) I would respect my personal**

**convictions** _1_ _2_ _3_ _4_ _5_ _6_

**i) I would be anxious about surgery** _1_ _2_ _3_ _4_ _5_ _6_

**j) I would be worried about inefficacy of**

**the drug tested** _1_ _2_ _3_ _4_ _5_ _6_

**k) I think that research only benefits**

**to the doctor’s career** _1_ _2_ _3_ _4_ _5_ _6_

**Question 7 : If you could receive financial compensation, would you be more willing to participate?**

certainly probably indifferent probably not certainly not no opinion

_1_ _2_ _3_ _4_ _5_ _6_

**Question 8 : If participating in the study required commuting to the hospital, would you decline to participate?**

certainly probably indifferent probably not certainly not no opinion

_1_ _2_ _3_ _4_ _5_ _6_

**Question 9 : If the study required blood tests, would you decline to participate?**

certainly probably indifferent probably not certainly not no opinion

_1_ _2_ _3_ _4_ _5_ _6_

**Question 10 : If the study required randomly assigned treatment, would you decline to participate?**

certainly probably indifferent probably not certainly not no opinion

_1_ _2_ _3_ _4_ _5_ _6_

**Question 11 : If the study required a treatment that was prescribed by the study and not by your doctor, would you be willing to participate?**

certainly probably indifferent probably not certainly not no opinion

_1_ _2_ _3_ _4_ _5_ _6_

**Question 12 : If the study involved a placebo (inactive substance), would you refuse to participate?**

certainly probably indifferent probably not certainly not no opinion

_1_ _2_ _3_ _4_ _5_ _6_

**Question 13 : Many clinical trials are double-blinded studies. This means that neither doctors nor the participants are aware which participants receive the test drug and which receive the placebo. Would you consider this a reason to decline participation?**

certainly probably indifferent probably not certainly not no opinion

_1_ _2_ _3_ _4_ _5_ _6_

**Question 14 : You will find below 6 vignettes in the field of anesthesia. Read them carefully and answer the question: "If you were the patient who was invited to participate, would you accept or refuse to participate in this study?"**

**Clinical Trial A: The objective of the study is to assess the effectiveness of a new drug not currently available on the market**

I would I would I don’t know I would I would

certainly accept probably accept if I would accept probably refuse certainly refuse

_1_ _2_ _3_ _4_ _5_

**Clinical Trial B: The purpose of the study is to assess the effectiveness of a new dosage of a well-established medication.**

I would I would I don’t know I would I would

certainly accept probably accept if I would accept probably refuse certainly refuse

_1_ _2_ _3_ _4_ _5_

**Clinical Trial C: The purpose of the study is to assess the effectiveness of a new indication of an established medication.**

I would I would I don’t know I would I would

certainly accept probably accept if I would accept probably refuse certainly refuse

_1_ _2_ _3_ _4_ _5_

**Clinical Trial D. The purpose of the study is to assess more accurately side effects of a well-established medication.**

I would I would I don’t know I would I would

certainly accept probably accept if I would accept probably refuse certainly refuse

_1_ _2_ _3_ _4_ _5_

**Clinical Trial E. The aim of the study is to evaluate a new locoregional anesthesia technique (i.e., epidural or nerve block).**

I would I would I don’t know I would I would

certainly accept probably accept if I would accept probably refuse certainly refuse

_1_ _2_ _3_ _4_ _5_

**Clinical Trial F. The objective of the study is to assess the reliability of a new anesthetic monitoring device (i.e., blood pressure measurement, electrocardiogram).**

I would I would I don’t know I would I would

certainly accept probably accept if I would accept probably refuse certainly refuse

_1_ _2_ _3_ _4_ _5_

**Question 15 : Have you ever been invited to participate to a clinical study in any medical area?**

_1_ yes _2_ no _3_ I don’t know

If you answered “no” or “I don't know” to this question, go directly to question 20.

If you answered “yes” continue with question 16.

**Question 16 : Have you agreed to participate?**

_1_ yes _2_ no _3_ I don’t know

**Question 17 : Have you ever been invited to participate to a clinical study in the area of anesthesia care?**

_1_ yes _2_ no _3_ I don’t know

**Question 18 : Have you agreed to participate?**

_1_ yes _2_ no _3_ I don’t know

If you answered “no” or “don't know” to this question, go directly to question 20.

If you answered “yes” continue with question 16.

**Question 19 : How did you experience your participation in anesthetic study?**

very rather indifférent rather very no

positively positively negatively negatively opinion

_1_ _2_ _3_ _4_ _5_ _6_

**Thank you for your participation. Here are some more general questions:**

**Question 20**

**A) You are** _1_ female _2_ male

**B) How old are you** ? ……….years

**C) How do you live ?** (many possible responses)

_1_ As a couple (married or cohabiting)

_2_ Alone (single, widowed or divorced)

_3_ With children under 18

**D) What is your education?**

_1_ Basic education

_2_ Apprenticeship

_3_ Upper secondary education

_4_ Short cycle tertiary education

_5_ Bachelor or higher university degree

_6_ Other

**E) What is your professional activity?**

_1_ Full-time employment

_2_ Part-time employment

_3_ Working at home

_4_ Unemployed

_5_ Student

_6_ Retired or invalid

**F) Do you work or have you worked in health care?**

_1_ Yes, medical

_2_ Yes, other

_3_ No

**G) What is your health insurance?**

_1_ Basic insurance

_2_ Supplementary or mutual insurance (private or semi-private)

_3_ Complementary insurance for alternative medicine (e.g. homeopathy, herbal medicine, osteopathy, reflexology, Chinese medicine)

**H) Do you have chronic disease?** _1_ yes _2_ no

**I) Do you take medication?** _1_ yes _2_ no

**J) If you take medication regularly, which disease do you have? ……………………………………………………………………………………………………………………………………………………………………………………………………….………………………………………………………………………………………………………**

**K) Have you ever been hospitalized?** _1_ yes _2_ no

**If yes, how many times? ……………**

**L) Have you ever had anesthesia?** _1_ yes _2_ no

If you wish to make remarks or comments, do not hesitate to formulate them in the space below:

**………………………………………………………………………………………………………………………………………………………………………………………………………………………………………………………………………………………………………………………………………………………………………………………………………………………………………………………………………………………………………………………………………………………………………………………………………………………………………………………………………………………………………………………………………………………………………………………………………………………………………………………………………………………………………………………………………………………………………………………………………………………………………………………………………………………………………**

THANK YOU FOR YOUR PARTICIPATION

Once completed, please insert this questionnaire into the stamped envelope and post it in a SwissPost mailbox or HUG mailbox.

Contact :

Service d’Anesthésiologie-HUG

Secrétariat

Etude Attitude, Barrières et Motivations

Rue Gabrielle Perret-Gentil 6-1211 Genève 14

[guy.haller@hcuge.ch](mailto:guy.haller@hcuge.ch) [isabelle.pichon@hcuge.ch](mailto:isabelle.pichon@hcuge.ch) [patrick.huwiler@hcuge.ch](mailto:patrick.huwiler@hcuge.ch)

[beatrice.gil-wey@hcuge.ch](mailto:beatrice.gil-wey@hcuge.ch)
